# 5-Nitro-1,3-bis­(prop-2-yn­yl)-1*H*-1,3-benzimidazol-2(3*H*)-one

**DOI:** 10.1107/S1600536813016814

**Published:** 2013-06-26

**Authors:** Youssef Kandri Rodi, Khalid Misbahi, Abdelkrim El-Ghayoury, Leokadiya Zorina, El Mokhtar Essassi, Lahcen El Ammari

**Affiliations:** a Laboratoire de Chimie Organique Appliquée, Université Sidi Mohamed Ben Abdallah, Faculté des Sciences et Techniques, Route d’Immouzzer, BP 2202 Fès, Morocco; b Unité Chimie Inorganique, Matériaux et Interfaces (CIMI), Université d’Angers, CNRS UMR 6200, France; c Institute of Solid State Physics RAS, 142432 Chernogolovka MD, Russia; d Laboratoire de Chimie Organique Hétérocyclique, URAC 21, Pôle de Compétences Pharmacochimie, Université Mohammed V-Agdal, BP 1014 Avenue Ibn Batouta, Rabat, Morocco; e Laboratoire de Chimie du Solide Appliquée, Faculté des Sciences, Université Mohammed V-Agdal, Avenue Ibn Battouta, BP 1014 Rabat, Morocco

## Abstract

The title compound, C_13_H_9_N_3_O_3_, crystallizes with two identical but differently oriented mol­ecules in the asymmetric unit, the dihedral angle between the fused-ring systems of the two molecules being 64.39 (7)°. The two prop-2-ynyl chains are located on opposite sides of the mol­ecule and are nearly perpendicular to the fused ring plane, as indicated by the C—N—C—C torsion angles in the range 106.0 (3)–113.4 (3)°. In the crystal, the two mol­ecules are linked through C—H⋯O hydrogen bonds into dimers, which are subsequently linked by further C—H⋯O inter­actions, building a three-dimensional network.

## Related literature

For the biological activity of benzimidazole derivatives, see: Horton *et al.* (2003 ▶); Kim *et al.* (1996 ▶); Roth *et al.* (1997 ▶). For examples of benzimidazol-2-one derivatives, see: Ouzidan *et al.* (2011*a* ▶,*b* ▶,*c* ▶).
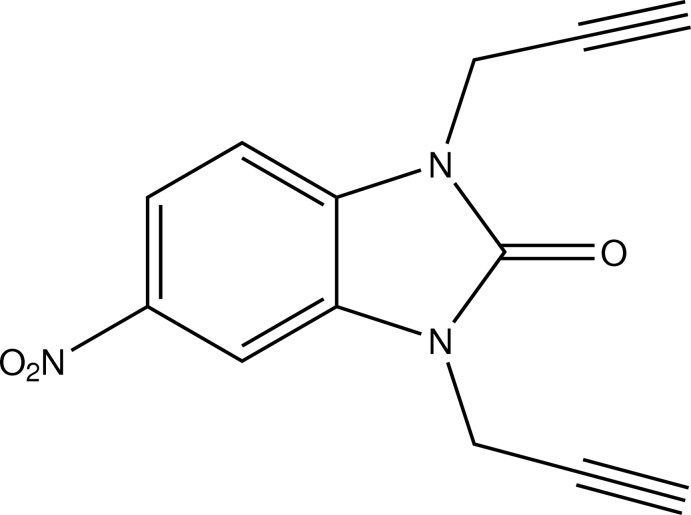


## Experimental

### 

#### Crystal data

C_13_H_9_N_3_O_3_
*M* *_r_* = 255.23
Orthorhombic, 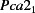

*a* = 20.0988 (16) Å
*b* = 4.2645 (3) Å
*c* = 28.669 (2) Å
*V* = 2457.3 (3) Å^3^
*Z* = 8
Mo *K*α radiation
μ = 0.10 mm^−1^
*T* = 150 K
0.48 × 0.2 × 0.13 mm

#### Data collection

Agilent Xcalibur (Ruby, Gemini) diffractometer
Absorption correction: multi-scan (*ABSFAC*; Agilent, 2012 ▶) *T*_min_ = 0.520, *T*_max_ = 1
16703 measured reflections
3233 independent reflections
2971 reflections with *I* > 2σ(*I*)
*R*_int_ = 0.032

#### Refinement

*R*[*F* ^2^ > 2σ(*F* ^2^)] = 0.047
*wR*(*F* ^2^) = 0.126
*S* = 1.05
3233 reflections
343 parameters
1 restraint
H-atom parameters constrained
Δρ_max_ = 0.48 e Å^−3^
Δρ_min_ = −0.29 e Å^−3^

### 

Data collection: *CrysAlis PRO* (Agilent, 2012 ▶); cell refinement: *CrysAlis PRO*; data reduction: *CrysAlis PRO*; program(s) used to solve structure: *SHELXS97* (Sheldrick, 2008 ▶); program(s) used to refine structure: *SHELXL97* (Sheldrick, 2008 ▶); molecular graphics: *DIAMOND* (Brandenburg, 2006 ▶); software used to prepare material for publication: *WinGX* (Farrugia, 2012 ▶) and *publCIF* (Westrip, 2010 ▶).

## Supplementary Material

Crystal structure: contains datablock(s) global, I. DOI: 10.1107/S1600536813016814/kj2228sup1.cif

Structure factors: contains datablock(s) I. DOI: 10.1107/S1600536813016814/kj2228Isup2.hkl

Supplementary material file. DOI: 10.1107/S1600536813016814/kj2228Isup3.cml

Additional supplementary materials:  crystallographic information; 3D view; checkCIF report

## Figures and Tables

**Table 1:** Hydrogen-bond geometry (Å, °)

*D*—H⋯*A*	*D*—H	H⋯*A*	*D*⋯*A*	*D*—H⋯*A*
C5—H5⋯O6^i^	0.95	2.43	3.316 (3)	155
C8—H8*A*⋯O4^ii^	0.99	2.28	3.186 (3)	151
C11—H11*A*⋯O6^i^	0.99	2.45	3.257 (4)	139
C13—H13⋯O2^iii^	0.95	2.32	3.205 (4)	155
C21—H21*B*⋯O1^iv^	0.99	2.27	3.191 (3)	154
C24—H24*B*⋯O3	0.99	2.49	3.350 (4)	146
C26—H26⋯O5^i^	0.95	2.40	3.320 (4)	164

Symmetry codes: (i) 
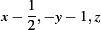
; (ii) 
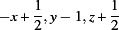
; (iii) 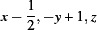; (iv) 
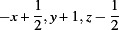
.

**Fig. 1.**
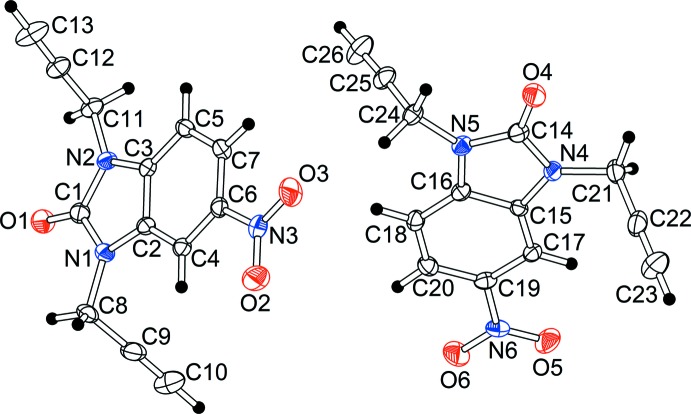
The molecular structure of the title compound with 50% probability displacement ellipsoids (molecule I, left; molecule II, right).

**Fig. 2.**
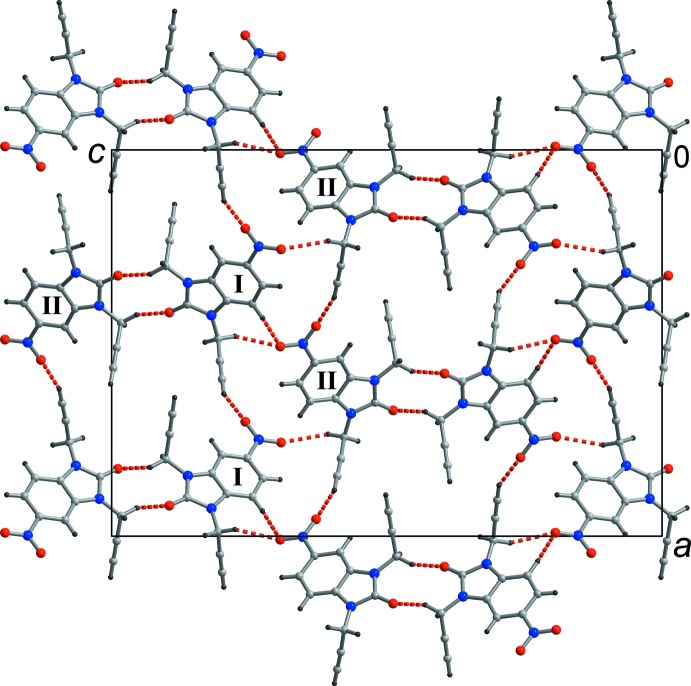
Packing diagram of the title compound viewed along the b–axis, showing the linkage between the molecule I (C1 to C13) and molecule II (C14 to C26). Hydrogen C—H···O bonds are shown as dashed lines.

## supplementary crystallographic information

### Comment

Benzimidazoles are very useful intermediates/subunits for the development of molecules of pharmaceutical or biological interest. Benzimidazole and its derivatives are an important class of bioactive molecules in the field of drugs and pharmaceuticals. Benzimidazole derivatives have found applications in diverse therapeutic areas including anti-ulcers, anti-hypertensive, anti-viral, anti-fungal, anti-cancers, (Horton *et al.*, 2003; Kim *et al.*, 1996; Roth *et al.*, 1997).

As a continuation of our research work devoted to the development of substituted benzimidazol-2-one derivatives (Ouzidan *et al.*, 2011*a*, 2011*b*), we report in this paper the synthesis of new benzimidazol-2-one derivative by action of propargyl bromide with 1*H*-benzo[*d*]imidazol-2(3*H*)-one in the presence of a catalytic quantity of tetra-n-butylammonium bromide under mild conditions to furnish two compounds: mono-substituted (Ouzidan *et al.*, 2011*c*) and di-substituted (Scheme 1).

The asymmetric unit of title compound, 1,3-Bis(prop-2-ynyl)-5-nitro-1*H*-benzo [*d*]imidazol-2(3*H*)-one, contains two molecules. Each of them is build up from two fused five- and six-membered rings liked to nitro group and to two prop-2-ynyl chains in opposite sides as shown in Fig. 1. The fused ring systems are almost planar, with the largest deviations from the mean planes being -0.005 (2) A° and 0.007 (3) A° for the C1 and C14 atom, respectively. In each molecule, the two prop-2-ynyl chains are nearly perpendicular to the fused ring plan as indicated by the torsion angles: C1–N1–C8–C9 = 111.8 (3)°; C1–N2–C11–C12 = 106.0 (3)°; C14–N4–C21–C22 = 113.4 (3)° and C14–N5–C24–C25 = 109.8 (3)°. The fused ring system belonging to the first molecule makes dihedral angle of 64.39 (7) ° with that of the second molecule. The difference between the two independent molecules lies in the crystallographic environment of each in addition to their orientations in the crystal. Indeed, in molecule I (C1 to C13), carbon C5 is involved in a C5—H5···O6 intermolecular hydrogen bond while in molecule II (C14 to C26) the corresponding carbon (C18 ) is not engaged in such a bond. In the crystal, the two molecules are linked through C8–H8A···O4 and C21–H21B···O1 hydrogen bonds in order to form dimers, which are linked together by the other C–H···O hydrogen bonds to build a three-dimensional network as shown in Fig.2 and Table 2.

### Experimental

To 5-nitro-1*H*-benzo[*d*]imidazol-2(3*H*)-one (0.2 g, 1.1 mmol), potassium carbonate (0.30 g, 2.2 mmol) and tetra-n-butylammonium bromide (0.07 g, 0.2 mmol) in DMF (15 ml) was added propargyl bromide (2.2 mmol). Stirring was continued at room temperature for 6 h. The salt was removed by filtration and the filtrate concentrated under reduced pressure. The residue was separated by chromatography on a column of silica gel with hexane/ethyl acetate (2/1) as eluent. Colourless crystals were isolated when the solvent was allowed to evaporate. Yield: 82%,mp: 415–417 K.

### Refinement

All H atoms could be located in a difference Fourier map. However, they were placed in calculated positions with C—H = 0.93 Å (aromatic), N—H = 0.86 and C—H = 0.97 Å (methylene) and refined as riding on their parent atoms with *U*_iso_(H) = 1.2 *U*_eq_ (C, N).

In the absence of significant anomalous scattering, the absolute configuration could not be reliably determined and thus 2580 Friedel pairs were merged.

### Crystal data

**Table:**

C_13_H_9_N_3_O_3_	*F*(000) = 1056
*M**_r_* = 255.23	*D*_x_ = 1.38 Mg m^−^^3^
Orthorhombic, *P**c**a*2_1_	Mo *K*α radiation, λ = 0.71073 Å
Hall symbol: P 2c -2ac	Cell parameters from 8380 reflections
*a* = 20.0988 (16) Å	θ = 1.8–29.7°
*b* = 4.2645 (3) Å	µ = 0.10 mm^−^^1^
*c* = 28.669 (2) Å	*T* = 150 K
*V* = 2457.3 (3) Å^3^	Prism, colourless
*Z* = 8	0.48 × 0.2 × 0.13 mm

### Data collection

**Table:**

Agilent Xcalibur (Ruby, Gemini) diffractometer	3233 independent reflections
Graphite monochromator	2971 reflections with *I* > 2σ(*I*)
Detector resolution: 10.4752 pixels mm^-1^	*R*_int_ = 0.032
ω–scan	θ_max_ = 29.7°, θ_min_ = 2.0°
Absorption correction: multi-scan (*ABSFAC*; Agilent, 2012)	*h* = −27→25
*T*_min_ = 0.520, *T*_max_ = 1	*k* = −5→5
16703 measured reflections	*l* = −38→36

### Refinement

**Table:**

Refinement on *F*^2^	Primary atom site location: structure-invariant direct methods
Least-squares matrix: full	Secondary atom site location: difference Fourier map
*R*[*F*^2^ > 2σ(*F*^2^)] = 0.047	Hydrogen site location: inferred from neighbouring sites
*wR*(*F*^2^) = 0.126	H-atom parameters constrained
*S* = 1.05	*w* = 1/[σ^2^(*F*_o_^2^) + (0.0875*P*)^2^ + 0.4146*P*] where *P* = (*F*_o_^2^ + 2*F*_c_^2^)/3
3233 reflections	(Δ/σ)_max_ = 0.002
343 parameters	Δρ_max_ = 0.48 e Å^−^^3^
1 restraint	Δρ_min_ = −0.29 e Å^−^^3^

### Special details

**Table:**

Geometry. All s.u.'s (except the s.u. in the dihedral angle between two l.s. planes) are estimated using the full covariance matrix. The cell s.u.'s are taken into account individually in the estimation of s.u.'s in distances, angles and torsion angles; correlations between s.u.'s in cell parameters are only used when they are defined by crystal symmetry. An approximate (isotropic) treatment of cell s.u.'s is used for estimating s.u.'s involving l.s. planes.
Refinement. Refinement of *F*^2^ against all reflections. The weighted *R*-factor *wR* and goodness of fit *S* are based on *F*^2^, conventional *R*-factors *R* are based on *F*, with *F* set to zero for negative *F*^2^. The threshold expression of *F*^2^ > 2σ(*F*^2^) is used only for calculating *R*-factors(gt) *etc*. and is not relevant to the choice of reflections for refinement. *R*-factors based on *F*^2^ are statistically about twice as large as those based on *F*, and *R*- factors based on all data will be even larger.

### Fractional atomic coordinates and isotropic or equivalent isotropic displacement parameters (Å^2^)

**Table:**

	*x*	*y*	*z*	*U*_iso_*/*U*_eq_	
O1	0.07911 (10)	−0.4511 (5)	0.39515 (8)	0.0308 (5)	
O2	0.29627 (10)	0.6066 (6)	0.25710 (8)	0.0407 (5)	
O3	0.24251 (12)	0.5944 (5)	0.19215 (8)	0.0398 (5)	
N1	0.15605 (10)	−0.1129 (5)	0.36150 (7)	0.0211 (4)	
N2	0.07187 (10)	−0.2738 (5)	0.31877 (7)	0.0205 (4)	
N3	0.24996 (12)	0.5152 (5)	0.23272 (10)	0.0248 (5)	
C1	0.09991 (12)	−0.2962 (6)	0.36290 (9)	0.0225 (5)	
C2	0.16290 (13)	0.0211 (5)	0.31805 (10)	0.0185 (5)	
C3	0.10972 (10)	−0.0835 (5)	0.29072 (8)	0.0185 (4)	
C4	0.21066 (11)	0.2190 (5)	0.30002 (8)	0.0198 (4)	
H4	0.2471	0.2922	0.3181	0.024*	
C5	0.10186 (14)	0.0045 (5)	0.24439 (10)	0.0208 (5)	
H5	0.0655	−0.0683	0.2262	0.025*	
C6	0.20185 (11)	0.3042 (5)	0.25370 (8)	0.0206 (4)	
C7	0.14964 (12)	0.2035 (6)	0.22587 (9)	0.0226 (5)	
H7	0.1466	0.2704	0.1943	0.027*	
C8	0.19954 (13)	−0.0708 (6)	0.40183 (9)	0.0257 (5)	
H8A	0.1781	−0.1625	0.4297	0.031*	
H8B	0.2060	0.1561	0.4076	0.031*	
C9	0.26444 (13)	−0.2195 (7)	0.39468 (10)	0.0317 (6)	
C10	0.31652 (16)	−0.3400 (10)	0.38963 (14)	0.0501 (9)	
H10	0.3586	−0.4374	0.3855	0.060*	
C11	0.01090 (12)	−0.4388 (6)	0.30644 (10)	0.0233 (5)	
H11A	0.0174	−0.5456	0.2761	0.028*	
H11B	0.0020	−0.6018	0.3302	0.028*	
C12	−0.04656 (12)	−0.2329 (6)	0.30314 (10)	0.0279 (5)	
C13	−0.09362 (15)	−0.0705 (8)	0.29930 (15)	0.0446 (8)	
H13	−0.1316	0.0605	0.2962	0.054*	
O4	0.32562 (11)	0.4775 (5)	−0.01141 (8)	0.0318 (5)	
O5	0.54368 (10)	−0.5661 (5)	0.12857 (8)	0.0394 (5)	
O6	0.49109 (12)	−0.5397 (6)	0.19373 (8)	0.0431 (6)	
N4	0.40313 (10)	0.1452 (5)	0.02253 (7)	0.0219 (4)	
N5	0.31780 (10)	0.3013 (5)	0.06502 (7)	0.0216 (4)	
N6	0.49818 (12)	−0.4715 (5)	0.15248 (10)	0.0248 (5)	
C14	0.34636 (12)	0.3236 (6)	0.02130 (9)	0.0225 (5)	
C15	0.41073 (12)	0.0130 (5)	0.06653 (10)	0.0176 (5)	
C16	0.35634 (11)	0.1154 (5)	0.09333 (8)	0.0189 (4)	
C17	0.45827 (11)	−0.1800 (5)	0.08478 (8)	0.0200 (4)	
H17	0.4950	−0.2527	0.0669	0.024*	
C18	0.34914 (13)	0.0292 (6)	0.13955 (10)	0.0215 (5)	
H18	0.3124	0.1011	0.1574	0.026*	
C19	0.44923 (11)	−0.2632 (5)	0.13144 (9)	0.0210 (4)	
C20	0.39648 (11)	−0.1631 (6)	0.15897 (9)	0.0229 (5)	
H20	0.3932	−0.2264	0.1907	0.028*	
C21	0.44756 (13)	0.1050 (6)	−0.01722 (9)	0.0264 (5)	
H21A	0.4536	−0.1217	−0.0234	0.032*	
H21B	0.4270	0.2003	−0.0452	0.032*	
C22	0.51236 (13)	0.2486 (7)	−0.00918 (10)	0.0317 (6)	
C23	0.56438 (16)	0.3678 (10)	−0.00310 (14)	0.0495 (8)	
H23	0.6064	0.4642	0.0018	0.059*	
C24	0.25700 (13)	0.4618 (6)	0.07741 (10)	0.0236 (5)	
H24A	0.2459	0.6155	0.0527	0.028*	
H24B	0.2640	0.5791	0.1068	0.028*	
C25	0.20067 (12)	0.2450 (6)	0.08344 (10)	0.0280 (5)	
C26	0.15561 (15)	0.0767 (8)	0.09000 (14)	0.0414 (8)	
H26	0.1190	−0.0598	0.0953	0.050*	

### Atomic displacement parameters (Å^2^)

**Table:**

	*U*^11^	*U*^22^	*U*^33^	*U*^12^	*U*^13^	*U*^23^
O1	0.0307 (10)	0.0354 (9)	0.0264 (12)	−0.0062 (8)	0.0009 (8)	0.0102 (8)
O2	0.0310 (10)	0.0498 (12)	0.0413 (13)	−0.0166 (10)	0.0000 (9)	0.0027 (10)
O3	0.0417 (12)	0.0488 (12)	0.0289 (11)	−0.0105 (10)	0.0098 (9)	0.0089 (10)
N1	0.0216 (9)	0.0238 (9)	0.0178 (9)	−0.0001 (8)	−0.0016 (7)	0.0026 (8)
N2	0.0171 (9)	0.0223 (9)	0.0221 (10)	−0.0024 (7)	−0.0015 (7)	0.0013 (7)
N3	0.0225 (10)	0.0241 (9)	0.0279 (14)	−0.0008 (8)	0.0069 (9)	0.0002 (8)
C1	0.0220 (11)	0.0234 (11)	0.0222 (12)	0.0015 (9)	0.0005 (8)	0.0014 (9)
C2	0.0174 (11)	0.0189 (9)	0.0193 (13)	0.0033 (8)	−0.0014 (9)	0.0001 (8)
C3	0.0159 (9)	0.0190 (10)	0.0206 (11)	0.0024 (8)	0.0020 (8)	−0.0008 (8)
C4	0.0177 (10)	0.0207 (10)	0.0209 (11)	0.0016 (8)	0.0003 (8)	−0.0024 (8)
C5	0.0224 (13)	0.0222 (10)	0.0179 (13)	−0.0014 (8)	−0.0021 (9)	−0.0024 (8)
C6	0.0195 (10)	0.0219 (10)	0.0204 (11)	0.0016 (8)	0.0029 (8)	−0.0024 (8)
C7	0.0251 (11)	0.0255 (10)	0.0171 (10)	0.0006 (9)	0.0002 (9)	−0.0010 (9)
C8	0.0281 (12)	0.0331 (12)	0.0158 (11)	−0.0028 (10)	−0.0055 (9)	0.0021 (9)
C9	0.0282 (13)	0.0429 (14)	0.0239 (12)	−0.0060 (11)	−0.0087 (10)	0.0057 (11)
C10	0.0288 (15)	0.070 (2)	0.051 (2)	0.0051 (16)	−0.0101 (14)	0.0029 (17)
C11	0.0184 (12)	0.0208 (10)	0.0309 (14)	−0.0019 (8)	−0.0017 (10)	−0.0008 (9)
C12	0.0195 (11)	0.0273 (11)	0.0368 (14)	−0.0047 (9)	−0.0009 (10)	−0.0001 (10)
C13	0.0211 (13)	0.0402 (15)	0.072 (2)	0.0006 (12)	−0.0075 (14)	−0.0002 (16)
O4	0.0327 (10)	0.0383 (10)	0.0245 (11)	0.0086 (8)	−0.0002 (8)	0.0102 (8)
O5	0.0267 (10)	0.0508 (12)	0.0408 (12)	0.0158 (9)	0.0005 (9)	0.0101 (10)
O6	0.0449 (13)	0.0591 (14)	0.0253 (11)	0.0161 (10)	−0.0056 (9)	0.0117 (10)
N4	0.0198 (9)	0.0282 (10)	0.0177 (9)	0.0022 (8)	0.0013 (7)	0.0044 (8)
N5	0.0195 (9)	0.0221 (9)	0.0232 (10)	0.0009 (7)	−0.0007 (7)	0.0043 (8)
N6	0.0234 (10)	0.0270 (10)	0.0240 (13)	0.0006 (8)	−0.0081 (9)	0.0032 (8)
C14	0.0201 (11)	0.0233 (11)	0.0239 (12)	−0.0003 (9)	−0.0012 (8)	0.0027 (9)
C15	0.0173 (10)	0.0200 (10)	0.0154 (12)	−0.0033 (8)	−0.0017 (9)	−0.0001 (8)
C16	0.0171 (10)	0.0173 (9)	0.0223 (11)	−0.0005 (8)	−0.0001 (8)	0.0005 (8)
C17	0.0160 (9)	0.0220 (10)	0.0220 (11)	−0.0010 (8)	0.0006 (8)	−0.0004 (8)
C18	0.0194 (12)	0.0233 (10)	0.0219 (14)	−0.0015 (8)	0.0036 (10)	0.0006 (9)
C19	0.0180 (10)	0.0195 (9)	0.0255 (11)	−0.0032 (8)	−0.0051 (8)	0.0034 (8)
C20	0.0255 (11)	0.0248 (11)	0.0185 (11)	−0.0035 (9)	−0.0010 (9)	0.0019 (9)
C21	0.0285 (12)	0.0306 (12)	0.0200 (12)	0.0027 (10)	0.0058 (9)	0.0019 (10)
C22	0.0272 (13)	0.0399 (14)	0.0279 (13)	0.0063 (11)	0.0087 (10)	0.0067 (11)
C23	0.0265 (15)	0.068 (2)	0.054 (2)	−0.0045 (15)	0.0038 (13)	0.0026 (17)
C24	0.0189 (11)	0.0226 (10)	0.0292 (14)	0.0044 (9)	0.0016 (10)	0.0016 (9)
C25	0.0204 (11)	0.0264 (11)	0.0372 (14)	0.0067 (10)	0.0006 (10)	−0.0042 (10)
C26	0.0232 (13)	0.0369 (15)	0.064 (2)	−0.0037 (12)	0.0085 (14)	−0.0073 (15)

### Geometric parameters (Å, º)

**Table:**

O1—C1	1.211 (3)	O4—C14	1.218 (3)
O2—N3	1.228 (4)	O5—N6	1.212 (4)
O3—N3	1.220 (4)	O6—N6	1.226 (4)
N1—C1	1.373 (3)	N4—C14	1.372 (3)
N1—C2	1.377 (3)	N4—C15	1.390 (3)
N1—C8	1.461 (3)	N4—C21	1.458 (3)
N2—C3	1.373 (3)	N5—C16	1.374 (3)
N2—C1	1.388 (3)	N5—C14	1.382 (3)
N2—C11	1.457 (3)	N5—C24	1.445 (3)
N3—C6	1.451 (3)	N6—C19	1.456 (3)
C2—C4	1.379 (3)	C15—C17	1.365 (3)
C2—C3	1.398 (3)	C15—C16	1.406 (4)
C3—C5	1.389 (4)	C16—C18	1.383 (4)
C4—C6	1.388 (3)	C17—C19	1.396 (3)
C4—H4	0.9500	C17—H17	0.9500
C5—C7	1.387 (4)	C18—C20	1.374 (4)
C5—H5	0.9500	C18—H18	0.9500
C6—C7	1.386 (3)	C19—C20	1.389 (3)
C7—H7	0.9500	C20—H20	0.9500
C8—C9	1.465 (4)	C21—C22	1.458 (4)
C8—H8A	0.9900	C21—H21A	0.9900
C8—H8B	0.9900	C21—H21B	0.9900
C9—C10	1.175 (4)	C22—C23	1.176 (5)
C10—H10	0.9500	C23—H23	0.9500
C11—C12	1.454 (4)	C24—C25	1.472 (4)
C11—H11A	0.9900	C24—H24A	0.9900
C11—H11B	0.9900	C24—H24B	0.9900
C12—C13	1.177 (4)	C25—C26	1.170 (4)
C13—H13	0.9500	C26—H26	0.9500
			
C1—N1—C2	110.2 (2)	C14—N4—C15	109.9 (2)
C1—N1—C8	122.6 (2)	C14—N4—C21	123.7 (2)
C2—N1—C8	127.2 (2)	C15—N4—C21	126.5 (2)
C3—N2—C1	110.46 (19)	C16—N5—C14	109.97 (19)
C3—N2—C11	127.6 (2)	C16—N5—C24	127.2 (2)
C1—N2—C11	122.0 (2)	C14—N5—C24	122.8 (2)
O3—N3—O2	123.2 (2)	O5—N6—O6	123.7 (2)
O3—N3—C6	119.0 (2)	O5—N6—C19	118.6 (3)
O2—N3—C6	117.8 (3)	O6—N6—C19	117.7 (3)
O1—C1—N1	128.1 (2)	O4—C14—N4	127.1 (2)
O1—C1—N2	126.4 (2)	O4—C14—N5	126.4 (2)
N1—C1—N2	105.5 (2)	N4—C14—N5	106.5 (2)
N1—C2—C4	131.5 (2)	C17—C15—N4	132.0 (2)
N1—C2—C3	107.3 (2)	C17—C15—C16	121.5 (3)
C4—C2—C3	121.2 (2)	N4—C15—C16	106.5 (2)
N2—C3—C5	131.1 (2)	N5—C16—C18	131.4 (2)
N2—C3—C2	106.5 (2)	N5—C16—C15	107.1 (2)
C5—C3—C2	122.5 (2)	C18—C16—C15	121.5 (2)
C2—C4—C6	115.5 (2)	C15—C17—C19	115.4 (2)
C2—C4—H4	122.3	C15—C17—H17	122.3
C6—C4—H4	122.3	C19—C17—H17	122.3
C7—C5—C3	116.9 (2)	C20—C18—C16	118.3 (2)
C7—C5—H5	121.6	C20—C18—H18	120.8
C3—C5—H5	121.6	C16—C18—H18	120.8
C7—C6—C4	124.5 (2)	C20—C19—C17	124.5 (2)
C7—C6—N3	117.2 (2)	C20—C19—N6	117.9 (2)
C4—C6—N3	118.3 (2)	C17—C19—N6	117.6 (2)
C6—C7—C5	119.6 (2)	C18—C20—C19	118.8 (2)
C6—C7—H7	120.2	C18—C20—H20	120.6
C5—C7—H7	120.2	C19—C20—H20	120.6
N1—C8—C9	111.6 (2)	C22—C21—N4	112.0 (2)
N1—C8—H8A	109.3	C22—C21—H21A	109.2
C9—C8—H8A	109.3	N4—C21—H21A	109.2
N1—C8—H8B	109.3	C22—C21—H21B	109.2
C9—C8—H8B	109.3	N4—C21—H21B	109.2
H8A—C8—H8B	108.0	H21A—C21—H21B	107.9
C10—C9—C8	179.0 (3)	C23—C22—C21	179.1 (4)
C9—C10—H10	180.0	C22—C23—H23	180.0
C12—C11—N2	113.1 (2)	N5—C24—C25	112.4 (2)
C12—C11—H11A	109.0	N5—C24—H24A	109.1
N2—C11—H11A	109.0	C25—C24—H24A	109.1
C12—C11—H11B	109.0	N5—C24—H24B	109.1
N2—C11—H11B	109.0	C25—C24—H24B	109.1
H11A—C11—H11B	107.8	H24A—C24—H24B	107.8
C13—C12—C11	178.1 (3)	C26—C25—C24	177.4 (3)
C12—C13—H13	180.0	C25—C26—H26	180.0
			
C2—N1—C1—O1	179.0 (3)	C15—N4—C14—O4	178.2 (3)
C8—N1—C1—O1	−2.1 (4)	C21—N4—C14—O4	−2.0 (4)
C2—N1—C1—N2	0.2 (3)	C15—N4—C14—N5	−0.4 (3)
C8—N1—C1—N2	179.2 (2)	C21—N4—C14—N5	179.4 (2)
C3—N2—C1—O1	−178.4 (2)	C16—N5—C14—O4	−177.9 (3)
C11—N2—C1—O1	1.1 (4)	C24—N5—C14—O4	0.7 (4)
C3—N2—C1—N1	0.3 (3)	C16—N5—C14—N4	0.7 (3)
C11—N2—C1—N1	179.8 (2)	C24—N5—C14—N4	179.3 (2)
C1—N1—C2—C4	−179.7 (2)	C14—N4—C15—C17	179.8 (2)
C8—N1—C2—C4	1.4 (4)	C21—N4—C15—C17	0.0 (4)
C1—N1—C2—C3	−0.7 (3)	C14—N4—C15—C16	−0.1 (3)
C8—N1—C2—C3	−179.6 (2)	C21—N4—C15—C16	−179.9 (2)
C1—N2—C3—C5	179.3 (2)	C14—N5—C16—C18	178.8 (2)
C11—N2—C3—C5	−0.2 (4)	C24—N5—C16—C18	0.2 (4)
C1—N2—C3—C2	−0.8 (3)	C14—N5—C16—C15	−0.8 (3)
C11—N2—C3—C2	179.8 (2)	C24—N5—C16—C15	−179.3 (2)
N1—C2—C3—N2	0.9 (3)	C17—C15—C16—N5	−179.4 (2)
C4—C2—C3—N2	−180.0 (2)	N4—C15—C16—N5	0.5 (3)
N1—C2—C3—C5	−179.2 (2)	C17—C15—C16—C18	1.0 (3)
C4—C2—C3—C5	0.0 (3)	N4—C15—C16—C18	−179.1 (2)
N1—C2—C4—C6	179.0 (2)	N4—C15—C17—C19	179.4 (2)
C3—C2—C4—C6	0.1 (3)	C16—C15—C17—C19	−0.7 (3)
N2—C3—C5—C7	180.0 (2)	N5—C16—C18—C20	180.0 (2)
C2—C3—C5—C7	0.0 (3)	C15—C16—C18—C20	−0.5 (3)
C2—C4—C6—C7	−0.1 (3)	C15—C17—C19—C20	0.0 (3)
C2—C4—C6—N3	179.3 (2)	C15—C17—C19—N6	179.4 (2)
O3—N3—C6—C7	0.1 (3)	O5—N6—C19—C20	178.4 (2)
O2—N3—C6—C7	179.8 (2)	O6—N6—C19—C20	−2.0 (3)
O3—N3—C6—C4	−179.3 (2)	O5—N6—C19—C17	−1.0 (3)
O2—N3—C6—C4	0.3 (3)	O6—N6—C19—C17	178.5 (2)
C4—C6—C7—C5	0.2 (4)	C16—C18—C20—C19	−0.2 (3)
N3—C6—C7—C5	−179.3 (2)	C17—C19—C20—C18	0.5 (4)
C3—C5—C7—C6	−0.1 (4)	N6—C19—C20—C18	−178.9 (2)
C1—N1—C8—C9	111.8 (3)	C14—N4—C21—C22	113.4 (3)
C2—N1—C8—C9	−69.5 (3)	C15—N4—C21—C22	−66.8 (3)
N1—C8—C9—C10	−144 (20)	N4—C21—C22—C23	−104 (25)
C3—N2—C11—C12	−74.5 (3)	C16—N5—C24—C25	−71.9 (3)
C1—N2—C11—C12	106.0 (3)	C14—N5—C24—C25	109.8 (3)
N2—C11—C12—C13	136 (10)	N5—C24—C25—C26	121 (7)

### Hydrogen-bond geometry (Å, º)

**Table:**

*D*—H···*A*	*D*—H	H···*A*	*D*···*A*	*D*—H···*A*
C5—H5···O6^i^	0.95	2.43	3.316 (3)	155
C8—H8*A*···O4^ii^	0.99	2.28	3.186 (3)	151
C11—H11*A*···O6^i^	0.99	2.45	3.257 (4)	139
C13—H13···O2^iii^	0.95	2.32	3.205 (4)	155
C21—H21*B*···O1^iv^	0.99	2.27	3.191 (3)	154
C24—H24*B*···O3	0.99	2.49	3.350 (4)	146
C26—H26···O5^i^	0.95	2.40	3.320 (4)	164

Symmetry codes: (i) *x*−1/2, −*y*−1, *z*; (ii) −*x*+1/2, *y*−1, *z*+1/2; (iii) *x*−1/2, −*y*+1, *z*; (iv) −*x*+1/2, *y*+1, *z*−1/2.
